# Cyclic pressure induced decellularization of porcine descending aortas

**DOI:** 10.1007/s10856-023-06723-5

**Published:** 2023-04-19

**Authors:** Barbara Messner, Maximilian Grab, Linda Grefen, Günther Laufer, Christian Hagl, Fabian König

**Affiliations:** 1 Department of Cardiac Surgery, Ludwig Maximilians University, Munich, Germany; 2 Cardiac Surgery Research Laboratory, Department of Cardiac Surgery, Medical University of Vienna, Vienna, Austria; 3 Chair of Medical Materials and Implants, Technical University Munich, Munich, Germany; 4 DZHK (German Centre for Cardiovascular Research), Partner site Munich Heart Alliance, Munich, Germany

**Keywords:** Porcine aorta, Pressure decellularization, Adventitial side, Intimal side, Detergent

## Abstract

The demand for decellularized xenogeneic tissues used in reconstructive heart surgery has increased over the last decades. Complete decellularization of longer and tubular aortic sections suitable for clinical application has not been achieved so far. The present study aims at analyzing the effect of pressure application on decellularization efficacy of porcine aortas using a device specifically designed for this purpose. Fresh porcine descending aortas of 8 cm length were decellularized using detergents. To increase decellularization efficacy, detergent treatment was combined with pressure application and different treatment schemes. Quantification of penetration depth as well as histological staining, scanning electron microscopy, and tensile strength tests were used to evaluate tissue structure. In general, application of pressure to aortic tissue does neither increase the decellularization success nor the penetration depth of detergents. However, it is of importance from which side of the aorta the pressure is applied. Application of intermittent pressure from the adventitial side does significantly increase the decellularization degree at the intimal side (compared to the reference group), but had no influence on the penetration depth of SDC/SDS at both sides. Although the present setup does not significantly improve the decellularization success of aortas, it is interesting that the application of pressure from the adventitial side leads to improved decellularization of the intimal side. As no adverse effects on tissue structure nor on mechanical properties were observed, optimization of the present protocol may potentially lead to complete decellularization of larger aortic segments.

**Graphical Abstract:**

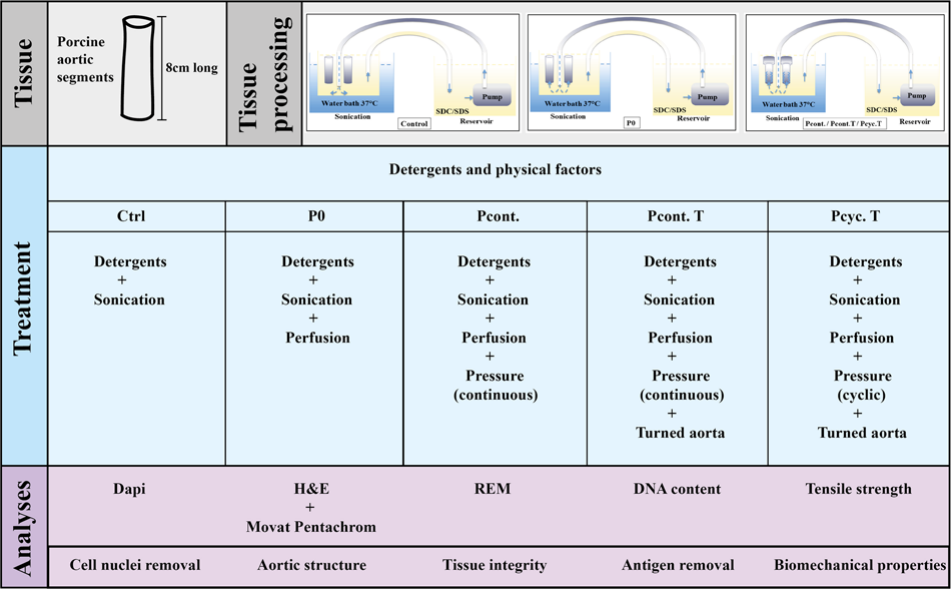

## Introduction

As cardiovascular diseases are still the number one cause of death globally [1], new surgical treatment strategies are needed to deal with the increasing number of patients. Therefore, the rapidly developing research area of tissue engineering (TE) is a promising approach, either in whole organ- or tissue-reconstructive surgery. Aside from newly developed biomaterials for damaged tissue replacement, decellularization of allogenic or xenogeneic vascular or heart tissue for biological scaffold preparation is an emerging field in tissue regeneration research [2–4]. One major advancement in cardiovascular tissue engineering was the creation of a complete decellularized heart to engineer a bio-artificial heart for transplantation [5]. Although remarkable progress has been achieved in the last decades after invention of the research area TE, improvement of present protocols as well as the invention of completely new strategies is needed, regarding decellularization techniques as well as the development of new polymers.

Based on the increasing numbers of patients with age-related cardiovascular diseases, like aortic aneurysm and myocardial infarction, vascular grafts with varying diameters for replacement are needed. Vascular tissue engineering approaches include the following techniques: decellularization, prostheses from natural polymers, prostheses from synthetic polymers, hybrid scaffolds, completely biological tissue engineered vascular grafts [6]. The application of synthetic grafts to replace large-diameter arteries results in high patency rates (e.g., the replacement of the aorta after aortic aneurysm surgery). In contrast, replacement of small-diameter arteries (<0.6 cm; coronary arteries after occlusion) with synthetic grafts showed a poor performance with occurrence of early thrombotic occlusion [7, 8]. Therefore, aside from the production and testing of polymeric grafts, decellularization of human and xenogeneic grafts represents a promising opportunity.

Aside from whole organ decellularization, various approaches were developed to decellularize vascular tissue needed in cardiovascular surgeries. Moreover, different tissue materials were used for decellularization, e.g., arterial and venous vascular walls, bovine, porcine, or equine pericardial tissue, intestinal submucosa and others [9]. Certainly, a large number of such decellularized materials are commercially available and used in clinical routine. Nevertheless, the performance of some of these products has been questioned lately (e.g., CorMatrix®) [10]. The recurring problems show how essential the development of new decellularization strategies still is.

The advantages of decellularized material serving as vascular grafts in TE are twofold: the possibility of using the natural architecture of the extracellular matrix and the resulting mechanical stability. Nevertheless, application of decellularized vascular grafts also revealed disadvantages such as adverse immune reactions, thrombotic events, and aneurysm formation resulting in performances only similar to synthetic grafts. Pioneering clinical studies have shown, that these grafts failed to meet expectations due to the lack of re-population of decellularized grafts with recipients own cells [11, 12]. As a consequence, projects where decellularized grafts were seeded with cells prior to implantation were developed. The in vitro bioreactor-mediated seeding with patients own bone-marrow derived endothelial and smooth muscle cells followed by implantation in a 10 year old girl resulted in a patency rate of 2 years, although the graft was narrowed after 9 month. In this case the graft was used as portal vein obstruction bypass [13]. Moreover, the efficacy of this strategy was also examined while using different animal models, concluding that prior cell seeding improves patency rates and confirms the fact that “in vivo” re-population is difficult to reach [14–16]. Nevertheless, also approaches using decellularized grafts which were prior implantation “conditioned” with endothelial cells were successfully tested [17].

Apart from difficulties to be repopulated by autologous cells, the decellularization of human or xenogeneic tissue is often incomplete, which can result in the induction of inflammatory reactions and consequently in early graft failure [18, 19]. Therefore, an adequate balance between complete decellularization and successful protection of extracellular matrix is desired. Methods used to decellularize vascular tissue are diverse. Mainly, the applied methods can be divided into the following groups: chemical agents, enzymes, and physical methods, whereby also combined protocols were tested [3, 4]. Based on these three main techniques, different decellularization protocols were put in place. It must be noted that the efficacy of each protocol depends on the tissue used and its composition (e.g., cell density, extracellular matrix composition, thickness) [4]. It seems that a combination of different methods would be the most efficient way. Aside from decellularization, removal of residual chemicals used for decellularization is also important. Therefore, extensive washing steps are needed to avoid a toxic environment for migrating cells [4]. Likewise important steps in generating vascular conduits and their application are the sterilization of decellularized grafts [3] as well as their preservation [20]. Both, sterilization as well as crosslinking has also undesirable effects affecting tissue stability and patency [3, 20]. Therefore, also these two important parts in vascular graft TE have to be improved for successful application in humans.

As mentioned above, replacement of aortic segments of large diameter and length e.g., after aortic aneurysm development, is performed using synthetic grafts (e.g., Teflon conduits) due to their longer patency rates and rapid availability. Aortic replacement is mainly needed in elderly patients due to the more frequent occurrence of such diseases. Nevertheless, regarding younger patients especially children, biological materials are preferred as they grow best with the rest of the body [21]. Biological scaffolds for reconstructive surgery are therefore also needed in a cardiovascular setting, especially for pediatric patients. Sources for the preparation of acellular extracellular matrix scaffolds are of human and/or of xenogeneic origin as for example porcine, based on their rapid availability. For cardiovascular tissue engineering, small diameter porcine arteries [22–25] as well as large diameter descending aortas [26–28] were used and subjected to decellularization by varying methods. In the course of these studies, different protocols and combinations of protocols were used in which decellularization efficacy and mechanical properties were analyzed. All of the mentioned studies either used detergents as sole chemical factor for decellularization or in combination with physical factors and enzymes. All of them showed a nearly complete decellularization and promising mechanical stability. Nevertheless, neither the application of pressure as a physical factor nor the usage of larger tissue pieces (if clearly stated piece size from 1.5 cm to 2 cm) are common to all protocols.

For that reason, the present study aims at analyzing the application of pressure in combination with SDC/SDS treatment using a specific apparatus applied to 8 cm long porcine descending aortic wall tissue.

## Material and methods

### Porcine tissue

Fresh porcine descending aortas were obtained from a local slaughter-house and segments of 16 cm length were cut out (aortic diameter ~2 cm, aortic wall thickness ~2 mm), whereby 8 cm pieces (cranial part) were used for decellularization and the caudal 8 cm pieces as native control tissue (to analyze the decellularization efficacy). After removal of connective tissue and fat residuals, cylindrical aortic segments were rinsed with PBS and stored for 16 h at 4 °C in PBS supplemented with 1%Streptomycin/Amphotericin/Penicillin.

### Decellularization procedure

**Fig. 1:**
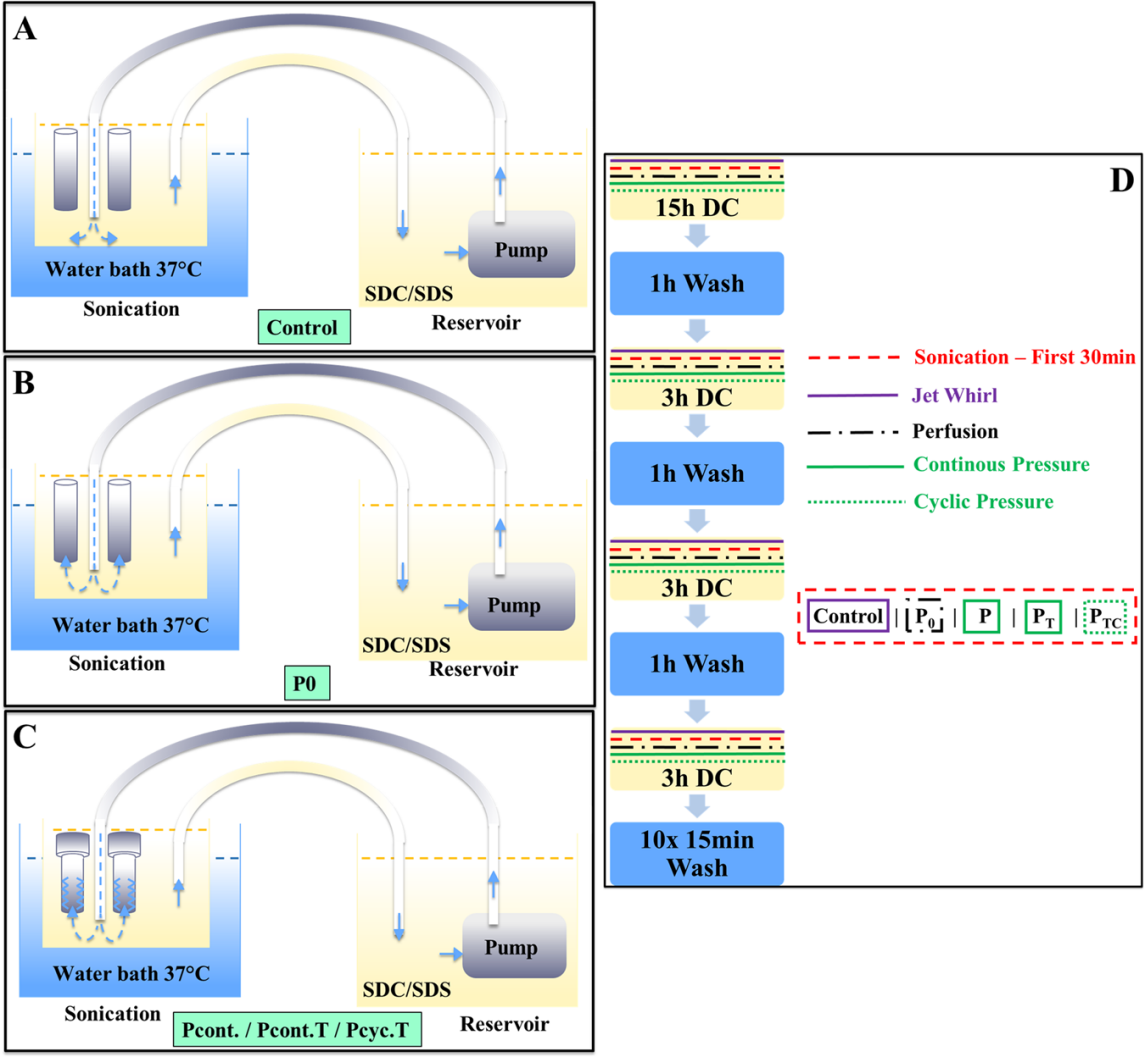
Schematic representation of the bioreactor and the used decellularization schemes for different incubations. In (**A**) the bioreactor setup for the Control (Ctrl) group is depicted, where the aortas are not fixed at the bottom but float in the decellularization solution. **B** Shows the setup for the P0 group (no pressure applied), where the aortic segments are fixed at the bottom of the bioreactor, enabling a bottom up perfusion of segments with decellularization solution. **C** Shows the setup for the Pcont. (application of continuous pressure), Pcont. T (application of continuous pressure to the turned aorta), and Pcyc. T (application of cyclic pressure to the turned aorta) groups. As in the P0 setting, the lower ends of the aortas are fixed to allow perfusion. In addition, these 3 settings have in common that a part is attached to the upper ends of the segments to slow down the flow of the decellularization solution that is built up by the pump and thus build up pressure on the aortic wall. **D** Shows the work flow for the different incubation groups

The decellularization solution used (for all treatment schemes), contained a combination of SDC (sodium deoxy cholate) and SDS (sodium dodecyl sulfate) at a concentration of 0.5%. Detergent incubation was performed at 37 °C (using a temperature controlled sonication water bath). As pump we used an Eden 155-80 W (20 l/min PfG GmbH). For details regarding the different treatment schemes please see Table 1. The basis for the different incubation schemes was treatment protocol G_3_ from Starnecker F et al. [29] (aortic segments of 4 cm length) and the design of a special bioreactor (Fig. 1). This specific protocol was chosen, since it results in a partial decellularization of the aortic vessel without damaging the tissue. Thus, this protocol is an ideal starting point to study the effect of pressure on decellularization efficiency.

**Table 1:** Overview of the experimental groups developed for this project.

	*Treatment groups*				
Procedure	Ctrl	P0	Pcont.	Pcont. T	Pcyc. T
Decellularization cycles (washing step after each DC cycle for 1 h)	15 h/	15 h/	15 h/	15 h/	15 h/
	3 h/	3 h/	3 h/	3 h/	3 h/
	3 h/	3 h/	3 h/	3 h/	3 h/
	3 h	3 h	3 h	3 h	3 h
Ultrasound (first 30 min of each cycle)	Yes	Yes	Yes	Yes	Yes
Jet whirl induced decellularization	Yes	No	No	No	No
Perfusion of aortic segments	No	Yes	Yes	Yes	Yes
Pressure induced decellularization by downregulation of flow lumen (continuous pressure application)	No	No	Yes	Yes	No
Turn of the aorta (adventitial side inside)	No	No	No	Yes	Yes
Cyclic pressure application (3 h of pressure in the 15 h DC cycle, 1 h in the 3 h DC cycle)	No	No	No	No	Yes
Final washing steps (10×)	Yes	Yes	Yes	Yes	Yes

Five different decellularization procedure were developed and the essential steps are shown in the Table

For the Ctrl group, the G3 protocol of Starnecker et al. [29]. was modified so that the aortic segments were loosely attached to the bottom of the bioreactor rather than floating in solution. In detail, decellularization was performed by using a jet whirl combined with a sonication treatment. Sonication treatment was performed only for 30 min at the beginning of each decellularization cycle (for details see Table 1) and the aortic segments were not actively perfused. As outlined in Table 1 the cyclic treatment scheme starts with 15 h of decellularization, followed by a 1 h washing step (with PBS), as well as alternate 3 times 3 h decellularization and 2 times 1 h wash. The final washing step is repeated 10 times for 15 min. Aortic walls of the second group (P0, no pressure application) were fixed at the bottom end of the bioreactor (only) and perfused with the decellularization solution from the bottom up (without fixing the aortas at the top). Tubular aortic walls of the third group (Pcont. continuous pressure applied) were fixed at the bottom as in group two, but the upper end also being fixed. In this group, the fixation on the upper end included a device ensuring a downregulation of the flow lumen to induce pressure of decellularization solution on the aortic wall. In contrast to group Pcont., the aortic tissues of the fourth group (Pcont. T, continuous pressure turned) was turned to apply the pressure not from the intimal side but from the adventitial side. Finally, in the last group (Pcyc T, cyclic pressure turned) aortic walls were turned and decellularized under cyclic pressure application (alternating 3 h of pressure and 1 h static treatment for 15 h and afterwards a 1 h long cyclic treatment).

### Tissue processing

After decellularization, aortic tissues were cut into pieces for the following analyses: for immunofluorescence and histological staining, tissue was fixed in 4.5% formalin for 24 h; tissues for scanning microscopic observations were fixed in 2.5% glutaraldehyde; tissue for DNA extraction were stored at −80 °C until extraction protocol is started; pieces for tensile strength experiments were punched out and stored in PBS at 4 °C and analyzed within 1 week. All samples were taken from the inner part of the aortic tube (not where aortas are fixed or immersed more efficiently in decellularization solution). Of note, for all analyses, tissue was taken from the center of the 8 cm segments and processed.

### Fluorescence staining for cell nuclei

After the decellularization procedure and fixation in formalin, tissue was dehydrated (by an ascending ethanol series andxylene) and subsequently embedded in paraffin. After preparation of 5 µm thick sections and deparaffinization (Roti-Histol, and a descending ethanol series with 100%, 96%, 70%), tissue sections were permeabilized with Aceton/Methanol (1:1) for 2 min and stained with DAPI solution (0.1 µg/ml) for 15 min. To reduce the autofluorescence of elastic fibers in aortic tissue, sections were incubated in Sudan black (0.3% in 70% Ethanol) for 2 min. Tissue sections were mounted using Ibidi mounting medium optimized for fluorescence microscopy. Subsequently images of stained tissue sections were taken using a fluorescence microscope (AxioObserver, Carl Zeiss, Germany). Decellularization efficacy was quantified by measuring the penetration depth (PD) form both sides (intimal as well as adventitial side). The PD was further subdivided into two areas: an area completely free of nuclei (decellularized area) and areas still containing cell nuclei residues (disintegrated area). Moreover, the PD is calculated in relation to total wall thickness and expressed as percent. Image acquisition and analysis was performed using a fluorescence microscope (AxioObserver, Calr Zeiss AG, Oberkochen, Germany). Equal exposure times were set for every sample and images are depicted as black and white images so that the cell nuclei are more visible.

### Histological staining

Fixed and dehydrated tissue sections were also used for histological staining. In detail, an H&E staining was performed to substantiate the DAPI results and to analyze potential structural changes due to the decellularization method. In order to do so, sections were deparaffinized (Roti-Histol, and a descending ethanol series with 100%, 96%, 70%) and stained with H&E according to the manufacturer’s instructions (Hemalum: Merck KGaA, Darmstadt, Germany; Eosin: Sigma-Aldrich Chemie GmbH, Germany). Images were taken using a Leica DM R microscope (Leica Microsystems GmbH, Wetzlar, Germany)

### Scanning electron microscopy (SEM)

Tissues were also prepared for SEM analyzes and taken images were used to visualize decellularization extent as well as to analyze tissue structure, e.g., affected extracellular matrix components as damaged elastic fibers which might have potential negative influences on tissue stability. Samples were fixed in an 864 mM glutaraldehyde solution supplemented with 1.5 mM hydrochloric acid and 52.79 mM sodium cacodylate trihydrate for at least 48 h at 4 °C. Dehydration of fixed samples was performed by immersion in an ascending ethanol series for 10 min (30%, 50%, 70%, 96% and 99.9%). Subsequently, samples were critical point dried (Leica Mikrosysteme Vertrieb GmbH, Wetzlar, GER) and gold coated (Leica Mikrosysteme Vertrieb GmbH). Image acquisition was performed using a scanning electron microscope (Zeiss Evo LS10, Carl Zeiss, Germany).

### DNA isolation

DNA isolation was performed with snap frozen aortic tissue using a DNA isolation kit for tissue (DNeasy Blood and Tissue Kit, Qiagen, Germany) according to the manufacturer instructions.

### Tensile strength tests

Aortic tissues were punched into strips of 5 cm length in circumferentially (circ.) and longitudinally (long.) orientation. Afterwards, thickness of specimens was measured. Both ends of the strip were sandwiched between the clamps using sand paper within a custom made device.

### Statistical analyses

Statistical analyses were performed using SPSS 21.0 software. Data are shown as mean ± SD and were tested for Gaussian distribution and subsequently differences were calculated using unpaired students *t* test. Values were considered as statistically significant if *p* < 0.05.

## Results

### Pressure-mediated decellularization within a bioreactor using detergents

In Fig. 1 the assembly of the bioreactor for the different treatment schemes (Fig. 1A–C) is depicted. Decellularization solution containing detergents (0.5% SDC/SDS) is pumped from the reservoir through a tube on the bottom of the flask containing the aortas (also completely filled with decellularization solution) to perfuse the aortas from the bottom up. Decellularization solution is circulating between the reservoir containing the pump and the reservoir containing the aortas. For group Ctrl, the lower ends of the aortas are loosely fixed at the bottom (the upper ends are unfixed). For the P0 group, the lower ends of the aortas are firmly fixed, the upper ends are also unfixed as in the Ctrl group (so perfusion of aortic segments from the bottom up without pressure application). For the group Pcont, both the lower and upper ends of the aortas are fixed. The upper end of each aorta is thereby connected to a small device, which narrows the lumen to increase the pressure on the inner side of the aortic wall. For the Pcon. T and Pcyc. T groups, the composition and attachment of the aortas is the same as in group Pcont., but for these two groups the aorta has been rotated so that the intimal side is on the outside and the adventitial side is on the inside. This applies pressure on the adventitial side of the aortas. The difference between the Pcon. T and Pcyc. T groups is that in Pcon. T the pressure is maintained constant for the decellularization period (15 h-3 h-3 h-3 h; Fig. 1D) and in the Pcyc. T group the pressure is applied cyclically (3 h of pressure and 12 h without pressure within the 15 h decellularization cycle and 1 h of pressure within each of the 3 h decellularization cycles).

### Influence of applied pressure on decellularization efficacy and aortic structure

**Fig. 2:**
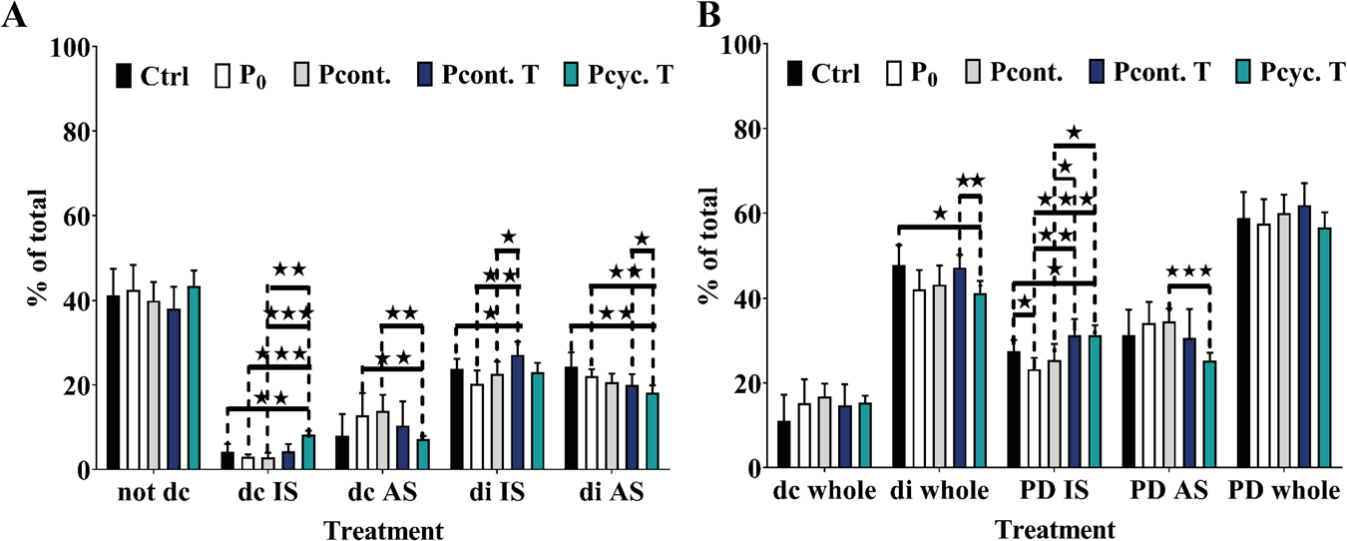
Quantitative and qualitative determination of penetration depth (PD). **A** Depicts the quantification of PD measurements of the five decellularization protocols. Measurements were taken for both the not decellularized area (not dc; innermost part of the aorta), and the decellularized area at the intimal side (dc IS) as well as adventitial side (dc AS). Moreover, PD measurements were also taken for disintegrated parts (intimal side = di IS and adventitial side = di AS) where cell nuclei residues are still present, shown in (**B**). Values are depicted as percent of control (in relation to total wall thickness). Analysis showed that none of the methods resulted in complete decellularization of aortic tissue. (Numbers per group: Ctrl *n* = 7; P0/Pcont./Pcont. T/Pcyc. T *n* = 5). * indicates *p* < 0.05, ** indicates *p* < 0.01, *** indicates *p* < 0.001

**Fig. 3:**
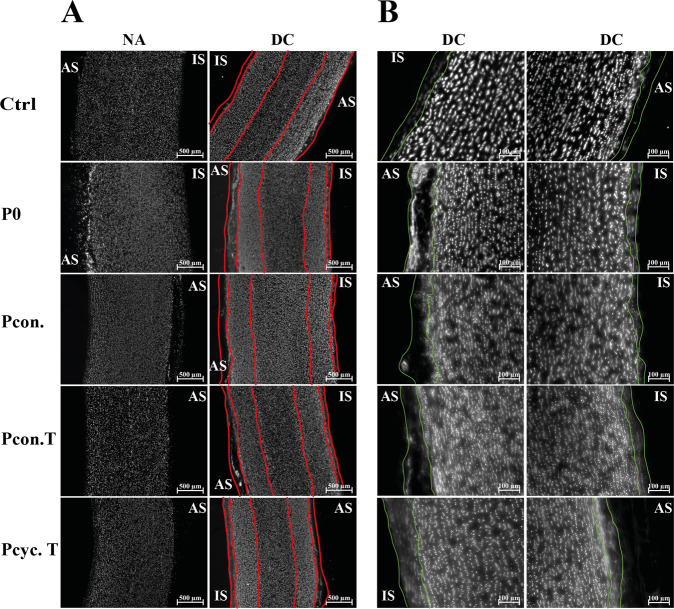
Representative images of DAPI stained aortic tissue. In (**A**) representative images of DAPI stained aortic tissues are shown (as black and white images; magnification ×5; scale bar is 500 µm). Red lines are labeling the three main areas: decellularized, disintegrated and not decellularized. Images in (**B**) were taken at a magnification of ×20 (scale bar is 100 µm). Green lines are depicting the completely decellularized area at the adventitial and intimal side. NA native tissue; DC decellularized tissue: IS intimal side; AS adventitial side

Figure 2A, B shows the quantification of decellularization extent. Using the DAPI staining, it was possible to quantify the areas completely free of cell nuclei (decellularized; dc), as well as areas where cell nuclei were disintegrated but not removed (disintegrated; di). These analyses were made at the intimal (IS) as well as the adventitial side (AS). Moreover, the penetration depth (PD) of decellularization solution was also calculated (calculated as the percentage of dc and di - for intimal side, adventitial side as well as in total). All of the calculations were made in correlation with total wall thickness (to compensate for individual thickness differences). In general, application of pressure (from both sides and also cyclic application) does not change decellularization efficacy and the penetration depth compared to the two control settings (Ctrl and P0). Interestingly, side dependent changes were observable: application of pressure from intimal side minimally (~10%) increases decellularization efficacy at the adventitial side and application of pressure form adventitial side minimally (~5%) increases decellularization efficacy at intimal side. Most effectively in regard to decellularization was the application of cyclic pressure (Pcyc. T) from the adventitial side (although simultaneously the amount of disintegrated nuclei is reduced compared to the controls). Similar results were observable regarding PD: cyclic application of pressure from adventitial side (Pcyc. T) increased PD at intimal side and reduced it at adventitial side. Of note, independent of the decellularization method used, the extent of complete decellularization was higher (often more than doubled) at the adventitial side compared to the intimal side. Figure 3 shows representative images of DAPI stained aortic tissue (illustrated in black and white). In Fig. 3A, images are shown at a magnification of ×5 and red lines are illustrating dc areas as well as di areas. Figure 3B shows images at a higher magnification of ×20 (intimal and adventitial side) and green lines are showing the decellularized areas.

### Aortic structure after pressure-mediated decellularization

**Fig. 4:**
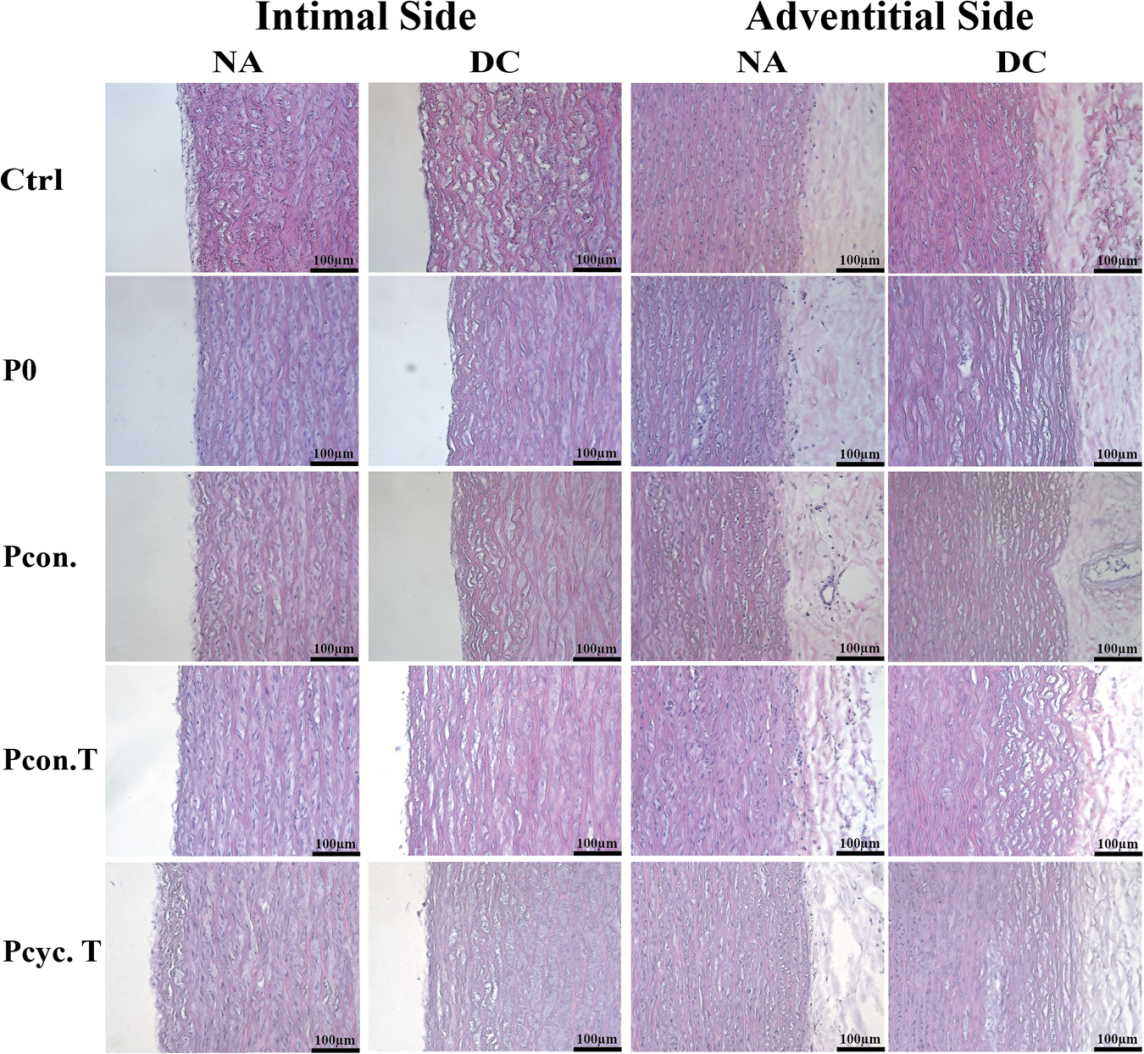
H&E staining of decellularized as well as native aortic tissue. Representative images of H&E stained aortic tissue, either native or decellularized, are shown. Images were taken at a magnification of ×20 (scale bar is 100 µm). Analysis of the images showed that none of the decellularization procedures resulted in impairment of the aortic structure. However, the loosening of the tissue in the areas of complete decellularization can be seen. This suggests that the endothelial cells and smooth muscle cells were completely removed in these areas. NA native tissue, DC decellularized tissue

**Fig. 5:**
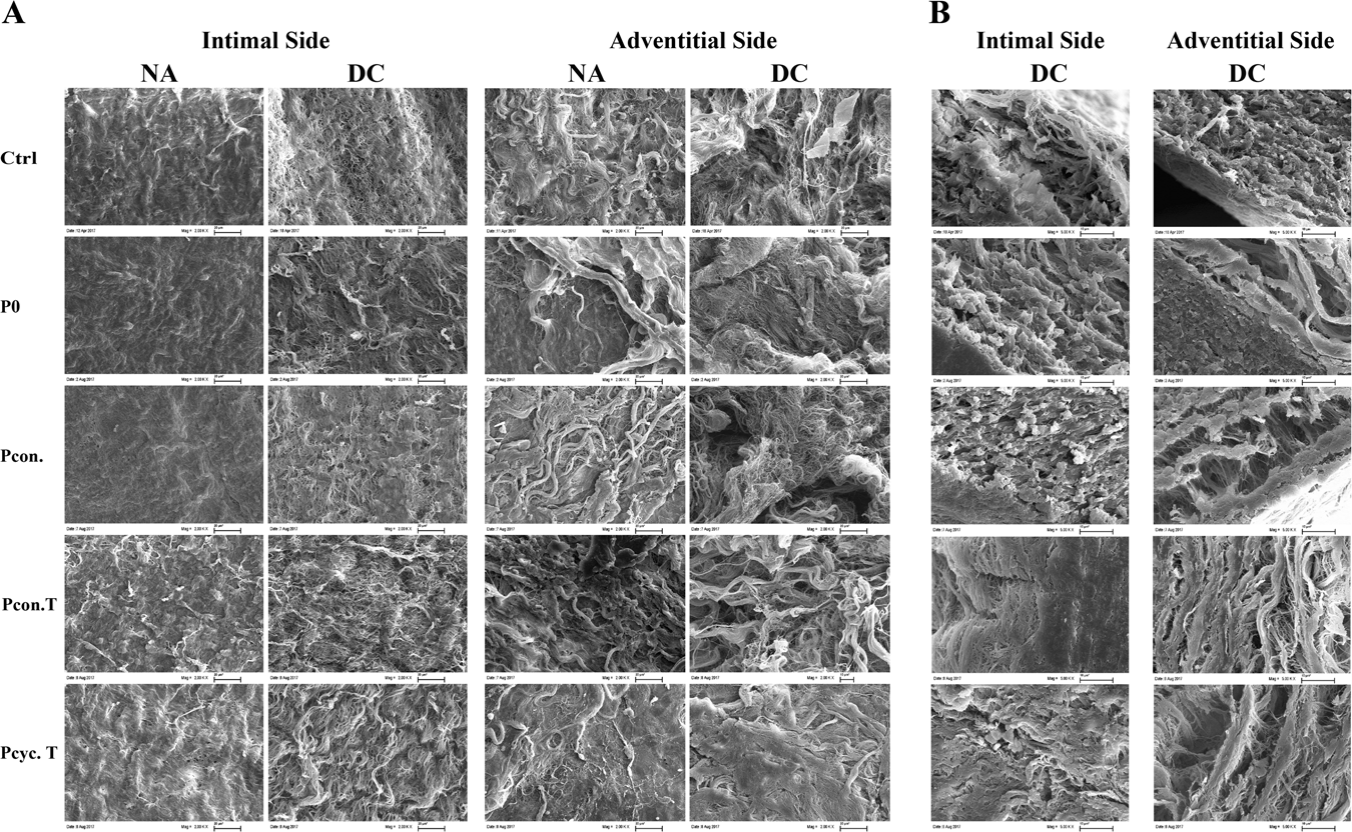
Scanning electron microscopic images of native as well as decellularized aortic tissue. **A** Shows images of the intimal as well as adventitial side of the aortic wall, either as native or decellularized tissue. Images were taken at a magnification of ×2.000 (scale bar is 20 µm). In (**B**) the lateral view on native or decellularized aortic tissue is depicted to show the aortic cross-section (intimal as well as adventitial side). Scanning electron microscopic images also show decellularization with no change in aortic structure. Images were taken at a magnification of ×5.000 (scale bar is 10 µm). NA native tissue, DC decellularized tissue

In addition, we also analyzed the structural appearance of aortic tissue after decellularization using H&E staining and scanning electron microscopic analyses. Figure 4 shows that none of the methods used does seriously affect aortic structure, since elastic fibers seem to be intact. The tissue appears loosened in the areas of complete decellularization (intimal and adventitial side). Moreover, areas completely free of cell nuclei and disintegrated cell nuclei were visible not only after DAPI staining but also in H&E stained tissue. Comparable results were provided by Movat pentachrome staining (Supplemental Fig. 1). None of the decellularization procedures resulted in impairment of the aortic structure. The extracellular matrix where the cells are embedded appeared to be intact, since in Movats pentachrome staining the loosened tissue is not as clearly visible as in the H&E stain. This is mainly due to the fact that pentachrome staining is used to mainly visualize the extracellular matrix. Representative images of this staining can be found in the online supplement (Supplemental Fig. 1). Aside from histological stainings, scanning electron microscopic images were taken to visualize the condition of the extracellular matrix (Fig. 5). Images of the intimal side (IS) and the adventitial side (AS) of native (NA) and decellularized (DC) aortas are shown in Fig. 5A. The intimal side of native tissues exhibited an intact endothelial monolayer. In contrast, the endothelial monolayer of decellularized tissue of all groups is absent, no endothelial monolayer is present, the extracellular matrix seems to be completely intact, and even though the matrix is looser in the Pcyc. T group (which was not observable on H&E stained tissues). Likewise, in contrast to the native tissue, SEM images of the adventitial side of the aorta showed no signs of cells or cell residues and no visible impairment of the extracellular matrix (Fig. 5A). In Fig. 5B, lateral images are shown to visualize the aortic media toward the intima as well as toward the adventitia. Areas free of cells are clearly visible as holes within a still intact extracellular matrix (most clearly visible at the adventitial side of group P0). Representative images are showing intact elastic fibers as well as the intact collagen network manifesting as thinner fibers. These images also displayed the low penetration depth.

### Effect of pressure-mediated decellularization on DNA content and biomechanical tissue properties

**Fig. 6:**
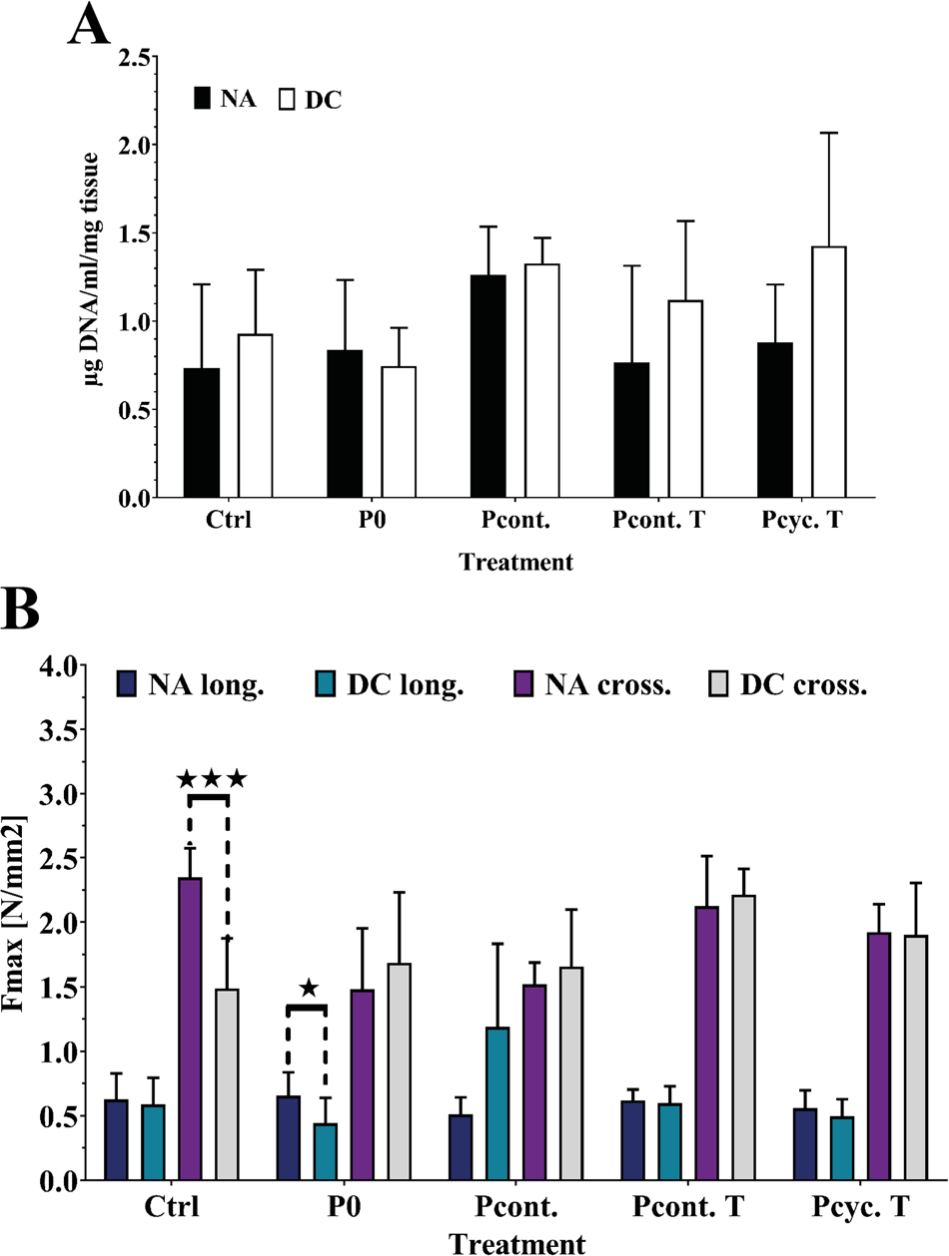
Determination of tissue DNA concentration as well as evaluation of the mechanical properties of decellularized vs native tissue. **A** Shows the results of DNA quantification within native as well as decellularized aortic tissue. No difference in DNA content was observed between native and decellularized tissue. In (**B**) results of tensile strength tests are depicted as Fmax [N/mm^2^] in circumferential and longitudinal orientation. Interestingly, this analysis revealed differences in in tensile strength of aortic tissue although decellularization was far from complete. (Numbers per group: Ctrl *n* = 7; P0/Pcont./Pcont. T/Pcyc. T *n* = 5). * indicates *p* < 0.05, *** indicates *p* < 0.001

As expected after quantification of PD, no reduction in the DNA content was observable after decellularization with each of the methods. Figure 6A shows that the applied technique for decellularization does not reduce the DNA content of descending aortic tissue, by none of the methods used. Since the PD was nearly the same within all tested groups, it is not really surprising that no significant change in DNA content was detectable. Although the used decellularization methods and specifically designed device do not improve decellularization efficacy, the tissue material was subjected to biomechanical tensile strength experiments. The detailed evaluation of tensile strength in circumferentially and longitudinally orientation are depicted in Fig. 6B. According to the structure of the aorta, it is not surprising that the Fmax is lower in longitudinally orientation compared to the circumferential. Decellularized aortas using the jet induced whirl combined with sonication treatment (Ctrl group) exhibited (in circumferential orientation) a significant lower Fmax compared to native tissue. In contrast, aortas decellularized in a fixed position without applying pressure (P0), the maximal force is reduced in longitudinally orientation compared to native tissue. These results are astonishing considering the lack of complete decellularization. In summary, compared to the corresponding native tissue, aortic tissue decellularized by applying pressure in the course of decellularization showed no alteration in maximal force, neither in circumferential nor in longitudinal orientation. However, such an effect could very well be present in the presence of impaired decellularization.

### Decellularization efficiency is also significantly dependent on the length of the aortic segments

**Fig. 7:**
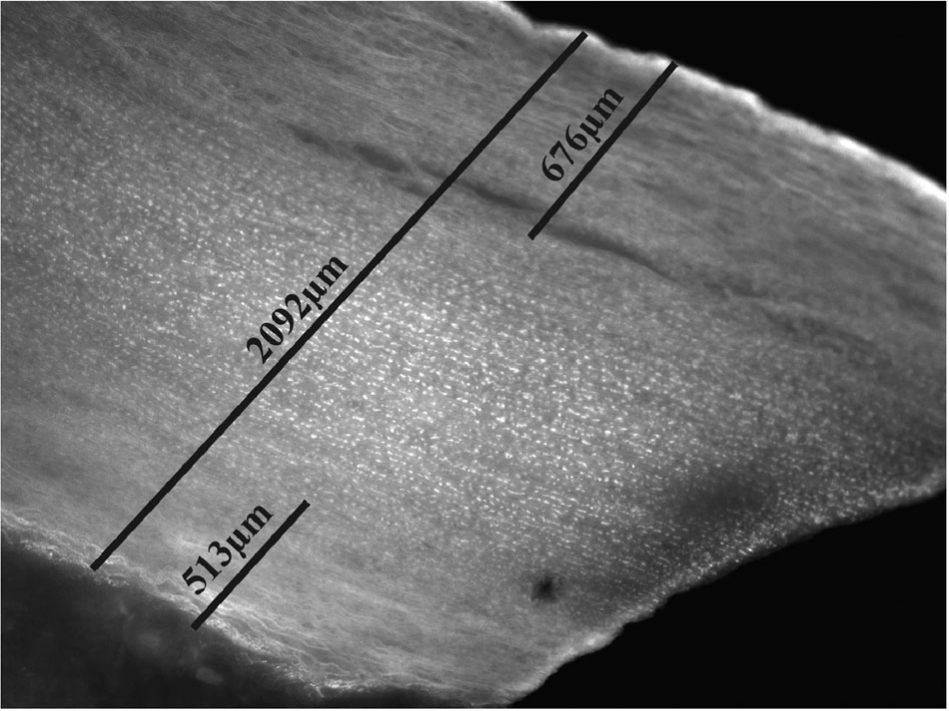
Decellularization of small aortic tissue piece using 0.5% SDC/SDC for 24 h. Figure 7 shows the representative image of a DAPI stained small sized aortic tissue piece (1.5 × 1.5 cm) after decellularization with 0.5% SDC/SDS for 24 h. Lines are indicating aortic wall diameter (2091.99 µm) and decellularization efficiency at the intimal side (676.42 µm) and adventitial side (513.67 µm). Decellularization of smaller aortic pieces significantly increases efficiency. Image was taken at a magnification if ×5

Based on the weak effect of SDC/SDS treatment on aortic tissue by executing the above described methods and the fact that published results are showing a higher effectivity in decellularization by using SDC/SDS we hypothesized that the effect depends on the length of the aortic segments. Therefore, we decellularized a 1.5 × 1.5 cm piece of aortic tissue for proof-of-concept using the same decellularization solution (0.5% SDC/SDS) and the same exposure times (15 h-3 h-3 h-3 h), to show that the decellularization protocol works. DAPI staining as well as quantification of PD of this piece revealed a complete decellularization of 32% (intimal side) and 24% (adventitial side) of aortic wall in comparison to 4% (intima side) and 8% (adventitia side) in 8 cm long aortic pieces and using the method Ctrl. Figure 7 shows the image of this piece stained using DAPI (black and white presentation). The lines show the total diameter of the aortic piece as well as the degree of decellularization on the intima and adventitia side. It should be noted here, however, that this test with the short piece was only carried out once.

## Discussion

The decellularization of human or xenogeneic tissue is an important research area in the development of the important field of TE, with the aim of meeting the increasing demand for organs and tissue replacement [30]. Nevertheless, until now no optimal strategy was developed to successfully decellularize and preserve natural tissue for vascular tissue engineering, as contradictory data were published or long term results are missing [31]. Accordingly, new strategies and protocols are needed without neglecting the tissue in terms of structure, function, and composition.

Aortic tissue is a thick-walled and compact material with a high elasticity to maintain and withstand blood pressure. The application of such decellularized material in reconstructive surgery requires (aside from complete decellularization) the preservation of extracellular matrix components for recipient’s cell in-growth, as well as maintenance of mechanical stability. Therefore, a combination of different decellularization protocols is advised. Decellularization protocols published so far include the use of chemical agents, enzymes, physical factors and a combination of those [3, 4]. In 2012 Zou et al. analyzed the decellularization efficacy of a combination of chemical and enzymatic agents using porcine descending aortas. They reported a complete decellularization using SDS and Triton X-100 combined with DNase and RNase alongside intact extracellular matrix composition and elastic properties [28]. As we have done in the course of the present study, Azhim et al. went a step further and combined the use of detergents with sonication as a physical factor to decellularize porcine aortic wall tissue. Their results confirmed that additional application of sonication significantly improves decellularization by increasing the penetration depth [26]. The ultimate aim of the above and the present study is to use physical factors in combination with chemical and enzymatic methods to increase the degree of decellularization, finally leading to a reduction in the required amount of detergents and enzymes [3]. An examination of physical factors as an alternative to detergent-based decellularization has already been carried out by Eichhorn et al. in their study. Comparative to SDS based decellularization of porcine aortic tissue, they analyzed the efficacy of high hydrostatic pressure, pressure shift freezing, and pulse electric fields. Although promising results, these three physical factors were not efficient as SDS treatment alone [32]. Of note, the authors stated that more detailed investigations regarding these methods are needed.

In the course of the present study, we tried to combine chemical agents with two different physical factors: sonication and pressure. The ultimate goal was to determine the impact of different pressure protocols on the DC efficiency while preserving the extracellular matrix. Therefore, a special apparatus was built to apply pressure on the 8 cm long aortic wall, and thus increasing the penetration depth of low concentrated SDC/SDS solution. In accordance to the original protocol, it was not possible to decellularize the porcine aortic tissue samples fully [29]. While this result is not suitable for final application of the porcine aortae, it allowed the observation of the direct impact of the added pressure protocols on the DC efficiency. Although the different pressure application protocols were not able to increase PD, we nevertheless observed two important issues for decellularization of aortic tissue: (i) application of pressure from the luminal side of the aorta reduces PD extent on this side, but increases the effectivity on the opposite side, and (ii) application of cyclic pressure at the adventitial side increases the decellularization extent at the intimal side significantly compared to the Ctrl group. Based on these results we hypothesized that application of pressure on one side leads to a relaxation of the tissue and thus facilitates infiltration of decellularization solution at the opposite side. This situation is improved by the application of cyclic pressure. Of note, as we not used DNase or RNase as an additional enzymatic factor, we observed disintegrated parts where cell nuclei residues were still present.

Nevertheless the efficacy of our present protocol was very low in contrast to already published data [26, 28, 29, 32], and also in comparison to studies decellularizing human or porcine aortic valves including aortic wall and leaflets [33–35]. According to histological and scanning electron microscopic evaluations, none of the used protocols have a negative effect on the structure of the aortas as the elastic fibres and the collagen network appear to be intact. Accordingly, quantification of tissue DNA concentration revealed no significant reduction as compared to native tissue. Of note, tensile strength tests interestingly revealed a reduced maximal force of circumferential orientated native compared to decellularized tissue within the Ctrl group. Likewise, in the P0 group, the maximal force of longitudinal orientated decellularized tissue is significantly reduced compared to the native control. Therefore, we conclude that these treatments are negatively affecting the structural function of these aortas, already at this stage of decellularization extent. It therefore appears that an improvement of these two protocols is not recommended, as more aggressive decellularization could affect the function of the tissue to a much greater extent.

Finally, to demonstrate that the chosen protocol with detergents and ultrasound application is in principle suitable for decellularization of aortic tissue, a small piece of aorta was decellularized in the same setting (without pressure). The efficiency was much higher than with the 8 cm aortic piece. This shows the complexity of the issue, as it is easy to decellularize small tissue components, unlike long and complex tubular structures.

## Conclusion

The results of the study can be summarized as follows: we were able to show that the use of chemical and physical methods leads to decellularization, but only in small aortic pieces. The effect on pieces of 8 cm length is limited. The application of pressure does not significantly improve the decellularization efficiency, although it is interesting to note that intimal pressure increases the efficiency on the adventitia side. In conclusion, the herein presented protocols have to be improved to reach a more effective decellularization of such a complex structure as the aorta. The added application of intraluminal and especially cyclic pressure offer great potential in the development of a protocol that relies much less on chemical detergents, therefore preserving the mechanical integrity of the tissue. In addition, future DC setups should focus on the alternating application of intra- and extraluminal pressure to further increase efficiency.

## Supplementary Information

Online Supplement
