# Supplementary material for: Cyclic pressure induced decellularization of porcine descending aortas
Source: J Mater Sci Mater Med. 2023 Apr 19;34(5):19. doi: 10.1007/s10856-023-06723-5 (PMC10115674; doi:10.1007/s10856-023-06723-5)
Supplement: Supplementary file 1 — Online Supplement [file 10856_2023_6723_MOESM1_ESM.docx]

**Online Supplement to**

**Cyclic pressure induced decellularization of porcine descending aortas**

Short title: **Decellularization of porcine descending aortas**

**Messner B.^1,2^, Grab M.^1,3^, Grefen L.^1^ Laufer G.^2^, Hagl C.^1,4^, Koenig F.^1^**

^1^ Department of Cardiac Surgery, Ludwig Maximilians University, Munich, Germany

^2^ Cardiac Surgery Research Laboratory, Department of Surgery, Medical University of Vienna, Austria

^3^ Chair of Medical Materials and Implants, Technical University Munich, Munich, Germany

^4^ DZHK (German Centre for Cardiovascular Research), partner site Munich Heart Alliance, Munich, Germany

**Corresponding author:**

Assoc. Prof. Priv. Doz. Mag. Barbara Messner, PhD

Medical University of Vienna

Department of Cardiac Surgery, Cardiac Surgery Research Laboratory

Währinger Gürtel 18-20

A-1090 Vienna, Austria

Email: [barbara.messner@meduniwien.ac.at](mailto:barbara.messner@meduniwien.ac.at)

Phone: 0043-40400-1-69490

## Material and Methods

## Histological stainings

Fixed and dehydrated tissue sections were used for histological stainings. Sections of 5µm thickness were used for staining of Movats pentachrom (MPC, Morphisto GmbH) according to the manufacturer’s instructions.

## Result

Staining of aortic tissue with Movats pentachrom and image acquisition as well analysis revealed that none of the decellularisation procedure induced changes in aortic structure, e.g. loss of elastic fibers and extracellular matrix. Loosening of the tissue in the decellularized areas is not as evident here as in the H&E staining. This could be because Movat Pentachrome staining specifically indicates the extracellular matrix, which is obviously still present.


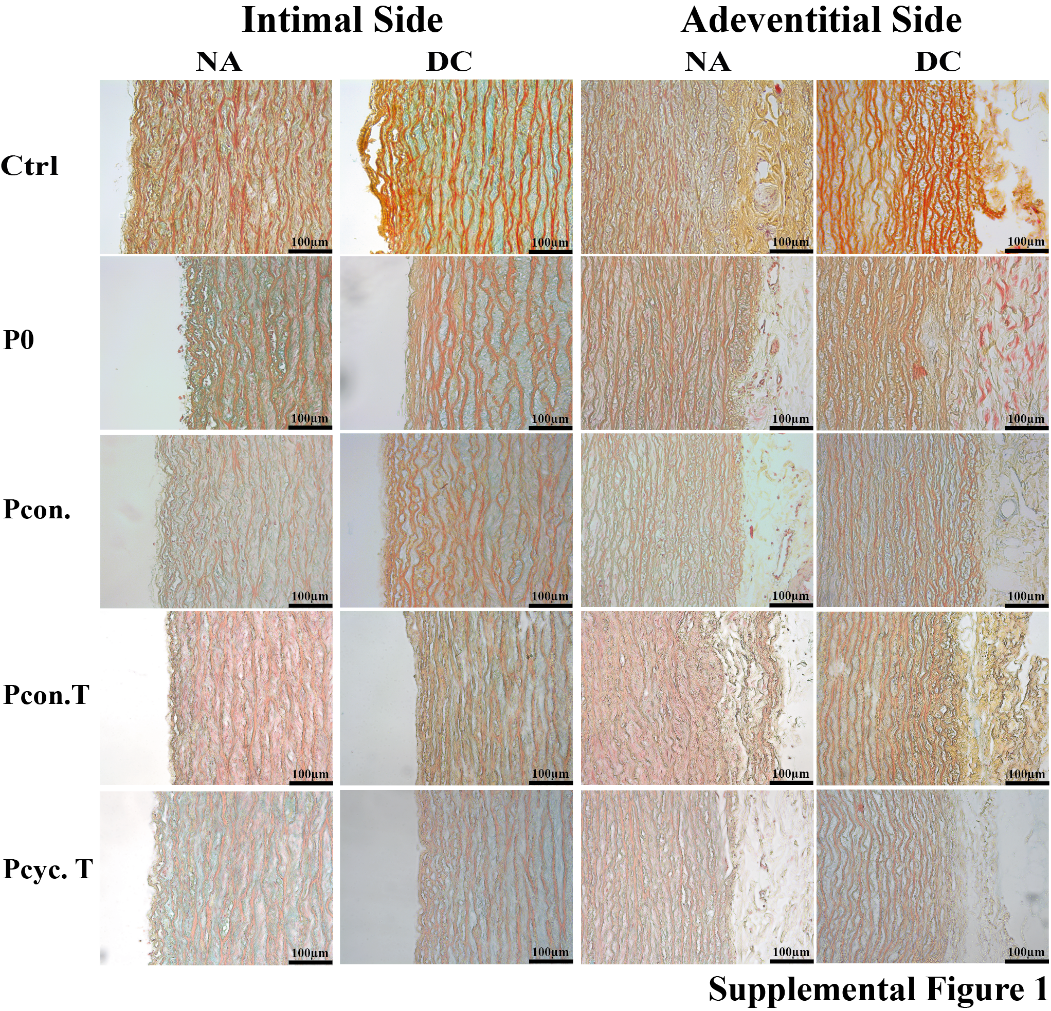


**Supplemental Figure 1:** **Movat pentachrom staining of decellularized as well as native aortic tissue.** Representative images of Movat Pentachrom stained aortic tissue, either native or decellularized, are shown. As for the analyses of H&E stained aortic tissue, also Movat pentachrom staining showed that none of the decellularization procedures resulted in impairment of the aortic structure. Loosening of the tissue in the areas of complete decellularization are only slightly visible, however not as clearly as in the H&E staining. Images were taken at a magnification of 20x. NA…..native tissue; DC…..decellularized tissue: IS…..intimal side; AS……adventitial side.
